# Homogeneous spectral spanning of terahertz semiconductor lasers with radio frequency modulation

**DOI:** 10.1038/srep44109

**Published:** 2017-03-08

**Authors:** W. J. Wan, H. Li, T. Zhou, J. C. Cao

**Affiliations:** 1 Key Laboratory of Terahertz Solid-State Technology, Shanghai Institute of Microsystem and Information Technology, Chinese Academy of Sciences, 865 Changning Road, Shanghai 200050, China

## Abstract

Homogeneous broadband and electrically pumped semiconductor radiation sources emitting in the terahertz regime are highly desirable for various applications, including spectroscopy, chemical sensing, and gas identification. In the frequency range between 1 and 5 THz, unipolar quantum cascade lasers employing electron inter-subband transitions in multiple-quantum-well structures are the most powerful semiconductor light sources. However, these devices are normally characterized by either a narrow emission spectrum due to the narrow gain bandwidth of the inter-subband optical transitions or an inhomogeneous broad terahertz spectrum from lasers with heterogeneous stacks of active regions. Here, we report the demonstration of homogeneous spectral spanning of long-cavity terahertz semiconductor quantum cascade lasers based on a bound-to-continuum and resonant phonon design under radio frequency modulation. At a single drive current, the terahertz spectrum under radio frequency modulation continuously spans 330 GHz (~8% of the central frequency), which is the record for single plasmon waveguide terahertz lasers with a bound-to-continuum design. The homogeneous broadband terahertz sources can be used for spectroscopic applications, i.e., GaAs etalon transmission measurement and ammonia gas identification.

Chemical sensing is one of the most important applications in the mid- and far-infrared (terahertz) wavelength ranges^1^,^2^,^3^ due to the abundant “fingerprints” (unique absorption lines) of various molecules in this frequency regime. The development of the terahertz spectroscopy is strongly dependent on the radiation sources. Although a time-domain spectroscopy system can cover a wide frequency range from 0.1 to 4 THz, it needs an optical pump and produces weak terahertz power on the level of dozens of milliwatts. Therefore, broadband and highly stabilized laser sources based on semiconductors are desirable for high-resolution spectroscopic applications.

Within the terahertz spectroscopic applications, the electrically pumped terahertz quantum cascade laser (QCL)^4^,^5^ is an ideal emitting source because of its high output power^6^ and octave-spanning spectra^7^,^8^. In principle, QCLs are inherently characterized by a narrow gain bandwidth and emission bandwidth due to the narrow linewidth of the inter-subband optical transitions. However, this can be overcome by properly designing the active region. Up to now, different approaches have been used for broadening the QCL gain bandwidth, such as the continuum-to-continuum transition^9^, dual upper-state to multiple lower-state transition^10^, multiple active region stacks^7^,^11^, and short-period superlattice structure^12^. Apart from the internal active region innovation, a technique employing external radio frequency (RF) modulation can also play an important role in bringing more longitudinal modes above the threshold and then broadening the emission spectra^13^,^14^,^15^,^16^. The RF modulation technique can actively mode-lock QCLs by modulating the laser current at the cavity roundtrip frequency (~*c*/2*ln*, where *c* is the speed of light, *l* is the laser cavity length and *n* is the refractive index). Therefore, in addition to the broad spectra, the mode spacing of the emission spectrum can be firmly locked at the frequency of the RF oscillator by external RF injection locking. Furthermore, by phase-locking the inter-mode beat note frequency with a repetition frequency of a femtosecond laser or another ultrastable oscillator, the QCL modes can be fully locked^17^,^18^,^19^. The fully locked lasers can then be used as a broadband emission source for high-resolution applications or a high-stability local oscillator reference.

In this work, using a hybrid active region structure, bound-to-continuum combined with resonant phonon extraction^20^, we achieve an ultralow threshold current density and broadband terahertz semiconductor QCLs. Due to the feature of an ultrlow threshold, we have the opportunity to investigate the spectral properties of a long-cavity (6 mm) terahertz QCL in continuous-wave (cw) mode. The inter-mode beat note signal is measured in both the frequency and time domains to characterize the frequency stability (or coherence) of the laser modes. Due to the long-cavity geometry that results in a smaller inter-mode beat note frequency and then stronger mode coupling to suppress the phase noise, we can efficiently modulate the laser and broaden the terahertz emission spectra utilizing a RF modulation technique. The modulated spectra can homogeneously span 330-GHz frequency range, which is approximately 8% of the central frequency. GaAs etalon transmission and ammonia gas (NH_3_) absorption measurements verify that the homogenous spectra of the QCL can be used in high resolution spectroscopic applications.

## Results

### Laser performance

**Figure 1:**
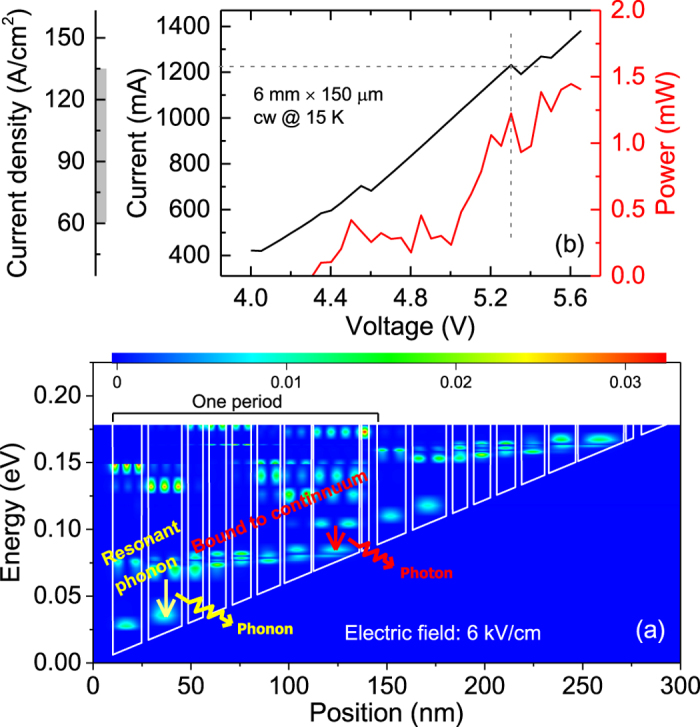
Active region design and light-voltage-current (*L*-*V*-*I*) characteristics of the terahertz QCL in cw mode. (**a**) Conduction band structure and the contour plot of the moduli squared of the wave functions of the hybrid active region at an electric field of 6 kV/cm. The layer sequence of one period, from right to left, is **4**.**1**/3.8/**1**/23.6/**1**/13.8/**2**.**1**/11.8/**3**.**1**/9.6/**3**.**1**/8.7/**3**.**1**/7.7/**3**.**1**/17.2/**3**.**4**/14.8 nm, where **Al**_0.25_**Ga**_0.75_**As** layers are in **bold**, GaAs in normal font, and the underlined layers are doped to 2 × 10^11^ cm^−2^. (**b**) Measured *L*-*V*-*I* characteristics of a 6-mm long and 150-*μ*m wide laser in cw mode at a heat sink temperature of 15 K. The vertical dashed line shows the voltage corresponding to the onset of the negative differential resistance (NDR) region, where the current and voltage are oscillating and the horizontal dashed line indicates the current density *J*_*max*_. The grey pattern on the “Current density” axis shows the laser dynamic range, defined as the current density range from the threshold to *J*_*max*_.

The active region of the terahertz QCL is a hybrid structure that combines the bound-to-continuum for photon emission with the resonant phonon design for fast depletion^20^,^21^ as shown in Fig. 1a. The radiative transitions take place from the bound state to the upper state of a continuum band emitting terahertz photons, corresponding to a frequency of 4.1 THz, as depicted by a red arrow. The electrons of the lower continuum band can be depopulated by the fast electron-phonon scattering, as depicted by a yellow arrow, releasing optical phonons. Information on the wafer growth and the device fabrication can be found in the Methods section.

In Fig. 1b, we show the measured light-voltage-current (*L*-*V*-*I*) characteristics of a 6-mm long and 150-*μ*m wide single plasmon waveguide laser recorded in cw mode at 15 K. Due to the hybrid active region design, the laser shows an ultralow threshold current density of ~60 A/cm^2^ (560 mA) and a wide dynamic range up to a *J*_*max*_ of 135 A/cm^2^ (1200 mA). Due to the feature of an ultralow threshold, we are able to operate the long-cavity laser in cw mode and then investigate the mode coherence and spectral properties in the following sections. At *J*_*max*_, the laser produces a more than 1 mW average power. Note that the power value shown in Fig. 1b represents the collected power without any corrections of the collection efficiency, window transmission, mirror reflection, and water absorption in the beam path. As the current is increased beyond 1200 mA, the current/voltage oscillation appears due to the misalignment of levels. Thus, in the inter-mode beat note and spectra analysis, we restrict the current to below 1200 mA. It is worth noting that by comparing Fig. 1b to the *L*-*V*-*I* curves of the shorter-cavity (~2.5 mm) QCLs shown in Supplementary Figs S2 and S4, the 6-mm long QCL shows less output power and non-continuous *L*-*V*-*I* characteristics. The possible reasons are (1) Non-uniform current injection along the device ridge across the active region due to the long cavity and not enough wire bonds on the top of the metal. (2) Imperfections in the device fabrication and the laser mounting process, which results in a performance degradation. Although the long laser shows worse electrical characteristic and a lower power generation, it demonstrates excellent spectral characteristics as will be shown in detail in the following text.

### Inter-mode beat note

Due to the resolution limit of commercial terahertz spectrometers, the frequency stability or phase noise of terahertz QCLs cannot be measured directly. However, the inter-mode beat note technique is a convenient method to indirectly characterize the frequency stability of Fabry-Pérot terahertz QCLs. When a laser is emitting in multimode operation, different longitudinal modes can beat with each other, and the fundamental beating frequency can normally be measured electrically or optically. The linewidth of the inter-mode beat note can be regarded as an indicator of the coherence of the laser. This indicates that less noisy terahertz modes give rise to narrow-linewidth inter-mode beat note spectra. In addition to the frequency domain characterization, the time trace of the beating signal can be also measured with a high-speed oscilloscope. The frequency- and time-domain results both reflect the coherence of terahertz QCLs. The experimental setup for the electrical inter-mode beat note measurement is shown in Supplementary Fig. S1.

**Figure 2:**
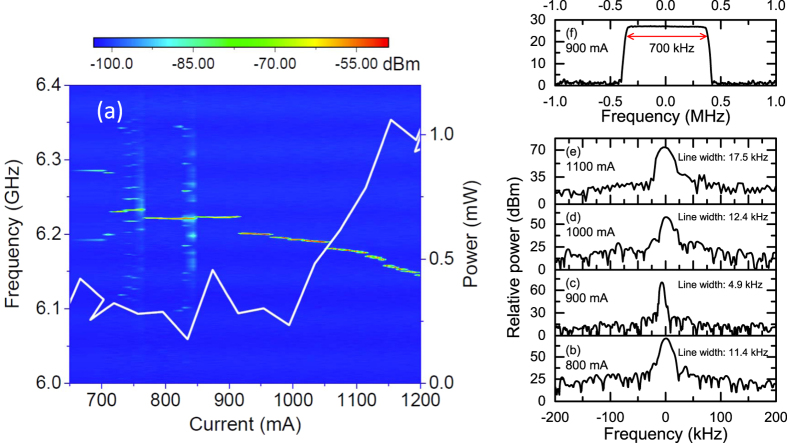
Electrical inter-mode beat note analysis for the 6-mm long terahertz QCL. (**a**) Electrical inter-mode beat note mapping. A 10-mA current step and 300-kHz resolution bandwidth (RBW) are used in this measurement. Each curve was recorded 20, and the results were averaged. The right y-axis shows the *L*-*I* curve for reference. (**b**–**e**) are the single-shot beat note spectra measured at 800, 900, 1000, and 1100 mA, respectively. The smallest RBW used for the linewidth measurement is 1 kHz. For clarity, the frequency of each spectrum is subtracted by the individual central frequency. (**f**) Beat note spectrum measured with the “Max Hold” function of the spectrum analyser at 900 mA. The time duration used for the “Max Hold” measurement is 3 minutes. For all the inter-mode beat note measurements, the QCL is running in cw mode, and the heat sink temperature is stabilized at 15 K.

Figure 2 shows the free-running electrical inter-mode beat note spectra of the 6-mm long terahertz QCL driven in cw mode at 15 K. In Fig. 2a, we show the beat note mapping together with the *L* − *I* curve of the laser. Each spectrum was measured in a trace average mode with an average number of 20. As shown in Fig. 2a, the inter-mode beat note frequency is approximately 6.2 GHz, close to the result obtained from the relation Δ*ν* = *c*/2*ln*. It can be seen that at a low drive current below 850 mA, the free-running beat note is characterized by broad or multiple-line spectra that indicate an inferior frequency stability or larger phase noise. A similar phenomenon was also observed in some other broadband terahertz QCLs^7^,^22^. However, as the current is increased beyond 850 mA, we always obtain single-line beat note spectra that show that at a higher current, the laser demonstrates better frequency stability. Due to the change in the refractive index with the laser current, we are able to see the red shift of the beat note frequency with the increasing drive current. To see the fine structure of the beat note spectra, we performed the high-resolution single-shot measurement shown in Fig. 2b–e for 800, 900, 1000, and 1100 mA, respectively. We can see that the linewidth of the single-shot beat note spectra is on the tens of kilohertz level. At 900 mA, we obtain the narrowest linewidth of 4.9 kHz, which indicates that the free-running laser is already close to the frequency comb operation. Although the single-shot spectra show narrow linewidths, on a larger time scale, the beat note frequency is still moving in a narrow frequency range. In Fig. 2f, the beat note spectrum measured with the “Max Hold” function of the spectrum analyser at 900 mA is shown. For a 3-minute duration, the beat note frequency spans 700 kHz.

**Figure 3:**
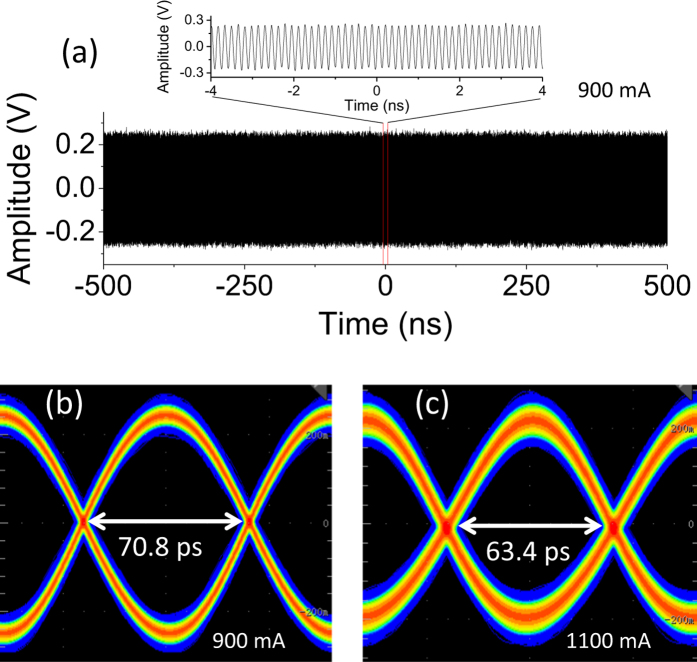
Time domain inter-mode beat note of the 6-mm long terahertz QCL. (**a**) Time trace of the inter-mode beat note signal recorded at 900 mA using a high-speed oscilloscope. The inset shows the zoomed-in figure with a time span of 8 ns. (**b**,**c**) Are eye diagrams constructed from the time traces measured at 900 and 1100 mA, respectively. In (**b**,**c**) the horizontal axes represent a time span of 160 ps.

In Fig. 3, we show the inter-mode beat note signal in the time domain employing a high-speed oscilloscope with the assistance of a low-noise amplifier (30 dB gain). Figure 3a plots the time trace obtained at 900 mA with a time span of 1 *μ*s. From the zoomed-in figure spanning 8 ns, we can see a clear sine shape time trace. The period of the trace is equal to the inverse of the inter-mode beat note frequency shown in Fig. 2. To quickly and intuitively assess the overall quality of a beat note signal in the full time scale, we construct eye diagrams, as shown in Fig. 3b,c, from the periodic sequence signal in the time domain. The wider the eye opens, the better the stability of the signal is. As examples, we show eye diagrams in Fig. 3b,c for 900 and 1100 mA, respectively. From 900 to 1100 mA, the eye width is reduced by ~10% from 70.8 to 63.4 ps which agrees well with the measurements in frequency domain shown in Fig. 2.

From the electrical inter-mode beat note analysis, we show that the 6-mm long terahertz QCL in free running demonstrates excellent mode coherence. At some drive currents, the free-running laser without any mode-locking technique implementation almost works as a comb. It is worth noting that this is not the case for short-cavity terahertz QCLs fabricated from the same wafer grown by molecular beam epitaxy. As shown in Supplementary Fig. S3, the measured electrical inter-mode beat note spectra from a 2.5-mm long terahertz QCL at most drive currents are broad. It is impossible to detect the time trace signal due to the decreased coherence. The coherence differences between the long- and short-cavity terahertz QCLs are analysed and explained in the following subsection.

### Terahertz spectra under RF modulation

In Figs 2 and 3, we show that the free-running laser already demonstrates favorable frequency stability. The single-line inter-mode beat note also indicates that the laser spectrum is naturally modulated by the cavity round trip frequency, and this modulation frequency falls in the microwave regime. Therefore, one can conveniently apply an external RF signal with a frequency close to the laser free-running beat note frequency onto the laser cavity to enhance the modulation. Consequently, we have two outcomes. First, due to the strong external modulation, more sidebands (or longitudinal modes) can overcome the loss and reach the threshold, and therefore the terahertz spectrum can be significantly broadened. Second, under external modulation, the mode spacing of the terahertz spectra can be exactly locked at the frequency of the RF synthesizer, and therefore, the frequency stability can be significantly improved.

**Figure 4:**
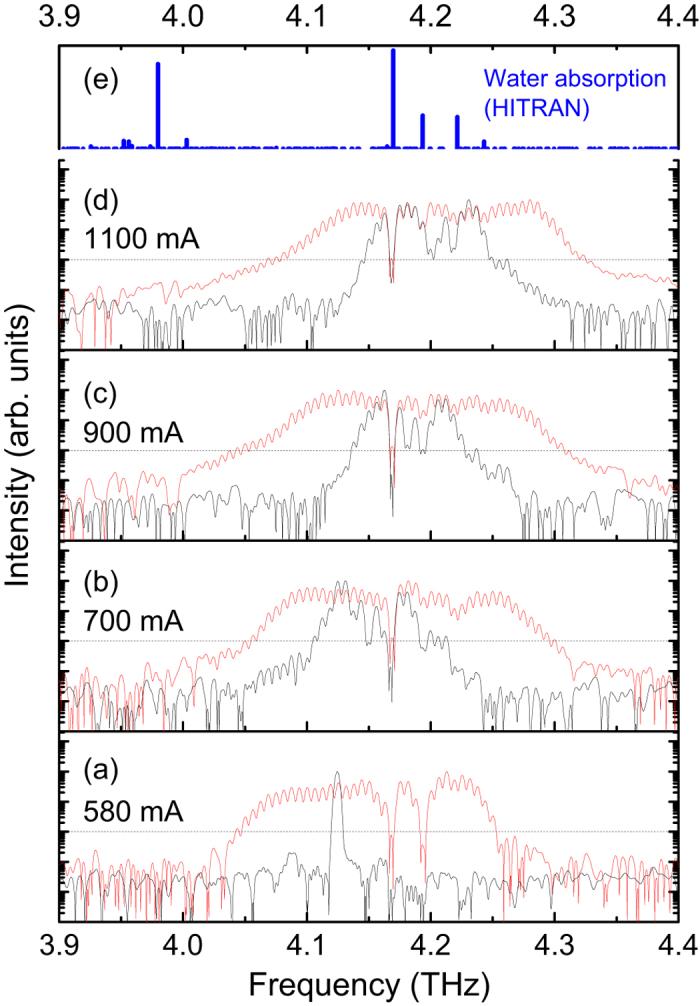
RF modulation effects on terahertz emission spectra. Emission spectra of the terahertz QCL measured at 580 (**a**), 700 (**b**), 900 (**c**), and 1100 mA (**d**). The black curves are taken when the laser is in free-running mode, and the red ones are the results obtained with the RF modulation. The horizontal dashed lines show the −20 dB level. (**e**) The water absorption lines in the frequency range from 3.9 to 4.4 THz extracted from the HITRAN database.

Figure 4 shows the RF modulation effects on the terahertz spectra of the 6-mm long cavity terahertz QCL at various drive currents. For comparison, the free-running terahertz emission spectra are also shown in black. For each case, the RF signal is injected at the peak frequency (approximately 6.2 GHz, see Fig. 2) of the individual free-running beat note spectrum with a RF power of 25 dBm. Note that due to the RF signal attenuation along the RF cable (see Supplement Fig. S6) and the reflection at the interface between gold wire and the laser facet caused by the impedance mismatch, the RF power that enters the laser cavity is much less than 25 dBm. For the sake of clarity, all the spectra shown in Fig. 4 are normalized to 1. The horizontal dashed lines show the −20 dB intensity attenuation level. At 580 mA (Fig. 4a) around the laser threshold, the free-running spectrum shows single-mode operation. However, after the RF modulation is applied, we see an obvious broadening in spectrum. Similar spectral broadening phenomena under external RF modulation are also clearly observed for higher currents (Fig. 4b–d). Employing the modulation technique, the mode spacing of the emission spectra is exactly locked at the injecting microwave frequency. It is worth mentioning that spectral resolution used for the Fourier transform infrared (FTIR) spectroscopy measurements is 0.1 cm^−1^ (3 GHz), and therefore the emission frequencies spaced by 6.2 GHz can be well resolved in Fig. 4. By counting the number of modes, we find that at 900 mA, the mode number is increased from 15 in free-running to 44 under RF modulation (only the modes with an intensity greater than −20 dB level are counted). At this drive current and the RF modulation condition, the emission spectrum continuously spans 260 GHz within the intensity attenuation of 20 dB. If we consider a −30 dB attenuation, the emission spectrum can continuously span 330-GHz frequency range (~8% of the central frequency) which is the record for single plasmon waveguide terahertz QCLs based on a bound-to-continuum active region.

Apart from the spectral broadening, we also obtain homogeneous terahertz spectra under RF modulation for the 6-mm long laser shown in Fig. 4. Furthermore, the modes at the water absorption frequencies can appear by exploiting the RF modulation technique. In Fig. 4e, we plot the water absorption lines extracted from the HITRAN database^23^ in the frequency range investigated here. There are 4 water absorption lines in the QCL frequency spanning range, and the strongest line is located at 4.17 THz. At some currents (Fig. 4b,c), the QCL mode at 4.17 THz can surmount the −20 dB level under RF modulation.

The efficient modulation observed in Fig. 4 benefits from the long-cavity geometry, which leads to a smaller inter-mode beat note frequency. First of all, the small beat note frequency results in dense longitudinal terahertz modes. As the mode spacing becomes smaller, the coupling between different modes becomes stronger, which can favour the mode coherence, as shown in Figs 2 and 3. We also measured the free-running inter-mode beat note spectra of a short-cavity (2.5 mm) laser from the same processed wafer and found that at most currents, we obtained broad beat note spectra (see Supplementary Fig. S3 for details). Therefore, the long-cavity laser is naturally more coherent, making it more efficient to actively modulate the long-cavity laser. In Supplementary Fig. S5, the modulation effect on a 2.47-mm long terahertz QCL is shown. Different from the efficient RF modulation for the 6-mm long QCL (see Fig. 4), the external RF modulation shows little influence on the emission spectra of the short-cavity QCL. However, considering the injection of the microwave signal into the laser cavity from the RF synthesizer, the 6 GHz signal is less lossy, and therefore, in principle, we can inject much more RF power into the cavity. As shown in Supplementary Fig. S6, we find that from one end to the other end of the RF cable, there is at least 3-dB difference in attenuation between 6 GHz and 15 GHz (2.5 mm cavity) injections. Indeed, in the experiment, we observed less efficient broadening in terahertz spectra for the short-cavity laser under RF modulation (see Supplementary Fig. S5).

**Figure 5:**
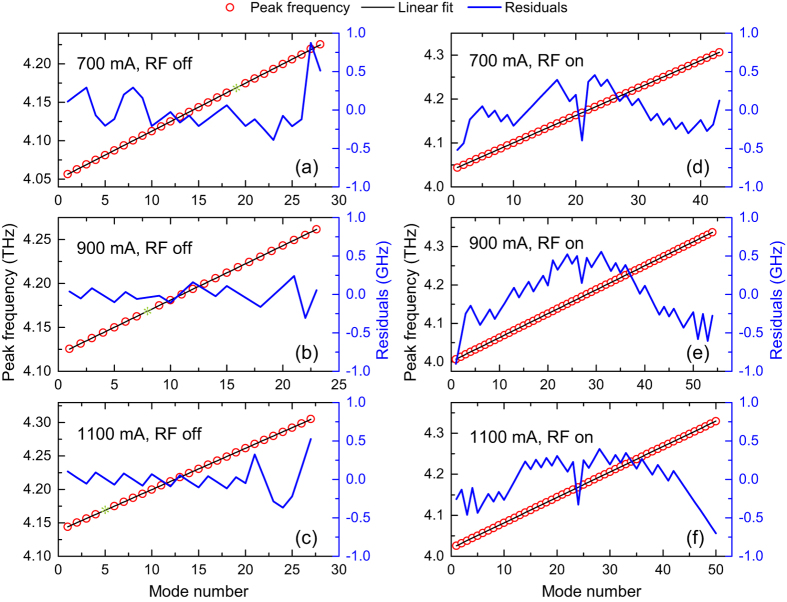
Dispersion measurements for the QCL at different drive currents and modulation conditions. Measured peak frequency (red circles) as a function of the mode number together with the linear fits (black lines). The right Y-axis shows the residuals from the measurements. Left column (**a**–**c**) are for free-running QCL at 700, 900 and 1100 mA, respectively, and right column (**d**–**f**) are for RF modulation at 700, 900 and 1100 mA, respectively. The green stars shown in (**a**–**c**) represent the missing peak frequency due to the strong water absorption at approximately 4.17 THz.

As has been elaborated in the literature, in QCLs, the total group velocity dispersion which causes a change in the mode spacing with frequency is the crucial point that should be well addressed for achieving broadband comb operation^7^,^24^,^25^. The narrow single-line beat note shown in Fig. 2 for the free-running terahertz QCL can, in principle, indirectly verify the small dispersion of the QCL gain medium in the frequency spanning range of 330 GHz. In Fig. 5, we directly analyse the dispersion by taking the Fourier transform of the interferograms with the zero-padding technique, which can be employed to accurately find the mode peak positions and then determine the mode spacing^26^. The left column of Fig. 5 shows the measured peak frequency as a function of the mode number (number 1 denotes the left-most visible longitudinal mode) together with the linear fits for the free-running QCL at different drive currents. The right column gives the results for the QCL under RF modulation at different drive currents. The right y-axis (blue) plots the residuals from the measurements, which is an indictor of the dispersion. From Fig. 5, we can see that for the free-running laser, the residuals are randomly distributed around 0. This indicates that the dispersion is zero or small, which is consistent with the beat note measurements shown in Fig. 2. Note that the larger residuals shown in Fig. 5a,c are probably due to the measurement uncertainty of the weak peaks in the tail of the spectra. Once the laser is modulated with the external RF signal, we see the broadening of the emission spectra or the increase in the mode numbers. Additionally, the distribution of residuals changes under RF modulation, i.e., we are able to clearly see some trends in Fig. 5d–f. Therefore, when the terahertz spectra become broad, the dispersion takes effect. Nevertheless, for all the cases presented in Fig. 5, the residuals are within ±1 GHz range. The small residuals actually indicate that the laser is demonstrating comb operation in both free-running and RF modulation modes.

### Terahertz spectroscopy

**Figure 6:**
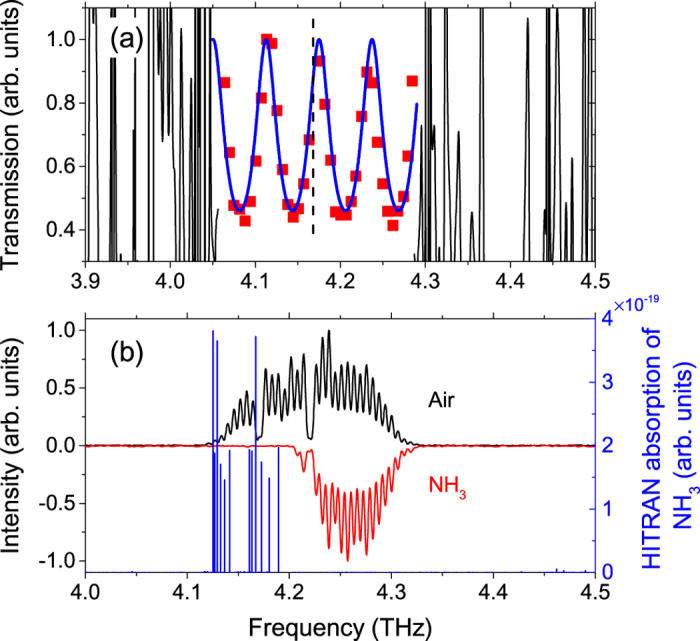
Terahertz spectroscopy using the homogeneous broadband terahertz QCL. (**a**) Transmission spectrum of a semi-insulating GaAs etalon sample (625 ± 25 *μ*m) using a homogeneous broadband terahertz QCL source under RF modulation at 6.1977 GHz and 25 dBm. The blue curve is a fit using a transfer matrix algorithm. The dashed vertical line shows the strong water absorption. (**b**) Spectral identification for the ammonia gas (NH_3_) using the homogeneous broadband terahertz QCL. The black spectrum is measured with a plastic bottle filled with air, and the flipped red spectrum is obtained with the same bottle filled with ammonia gas. Both spectra are normalized. The blue lines represent the absorption of ammonia gas given by the HITRAN database.

For the first proof of terahertz spectroscopy using the homogeneous broadband QCL with a 6-mm long resonant cavity, we measure the transmission of a double-side polished GaAs etalon sample with a nominal thickness of 625 ± 25 *μ*m using the QCL as the terahertz source. The measured transmission of the etalon, as shown in Fig. 6a (red squares), was obtained by taking the ratio of the peaks of the spectrum with the etalon to those measured without the etalon. As expected, the etalon shows a periodic transmission with frequency, and the period is measured to be 62.5 GHz. We also show the transmission fit for the etalon sample in Fig. 6a. Regarding the fit, we use a transfer matrix algorithm^27^ with fitting parameters of the etalon thickness *L* and the refractive index *n*. The values of the etalon thickness and refractive index obtained from the fitting algorithm are 659.5 *μ*m and 3.65, respectively. The computed thickness is in agreement with the nominal GaAs etalon thickness. Note that we show the direct intensity spectroscopy in Fig. 6a using the actively stabilized broadband QCL, and the spectral resolution is determined by the mode spacing of the QCL (or the injection frequency of RF synthesiser), which is 6.1977 GHz in this case.

Besides the etalon transmission measurement, we achieve the spectral identification for ammonia gas using the homogeneous broadband laser, as shown in Fig. 6b. The absorption of ammonia gas extracted from the HITRAN database is plotted as blue lines. It can be seen from Fig. 6b that once the bottle is filled with ammonia gas, the left part of the spectrum spanning from 4.1 to 4.2 THz is completely removed. Ideally, the absorption lines should be resolved. However, due to the fact that the gas inside the bottle is a mixture (ammonia gas, water vapor and air) rather than pure ammonia gas, and the complex gas mixture could broaden the absorption lines and form an absorption band, we finally observe in Fig. 6b that a block of spectrum is removed. Although the ammonia gas absorption lines cannot be resolved, this measurement can be used to identify the species of the gases.

## Discussion

We have shown that bound-to-continuum terahertz QCLs utilizing external RF modulation can produce homogeneous broadband spectra that continuously span 330 GHz (corresponding to 8% of the central frequency). The homogeneous broadband laser is successfully used for direct spectroscopic applications, i.e., transmission measurement of a GaAs etalon and the spectral identification of ammonia gas. Although the RF modulation technique is a robust tool to achieve homogeneous spectra for terahertz QCLs and a record frequency spanning 330 GHz for bound-to-continuum terahertz QCLs has been demonstrated in this work, practical implementations of terahertz spectroscopy in principle require even wider frequency coverage, e.g., octave-spanning spectra^7^, for various applications. Considering the ultrabroad octave-spanning spectra generated from terahertz QCLs, the index dispersion is the key factor and should be carefully solved.

The spectral resolution presented here is approximately 6.2 GHz, which is comparable to that of a commercial FTIR spectrometer. To further improve the resolution, the technology of dual-comb spectroscopy^28^,^29^ should be employed. In this work, the mode spacing of the longitudinal modes is actively locked by the external RF injection. However, the carrier is not locked, and therefore the modes with fixed spacing are moving in a small frequency range as an ensemble. By locking the carrier to an ultrastable oscillator, the QCL modes can be fully locked. Then the dual-comb spectroscopic technique can be used for ultrahigh resolution (MHz level) spectral measurements.

## Methods

### Sample growth and device fabrication

The QCL structure presented in this work is based on an Al_0.25_Ga_0.75_As/GaAs material system grown by the molecular beam epitaxy (MBE) on a semi-insulating GaAs (100) substrate. The growth starts with a 400-nm thick n-type GaAs bottom contact layer doped to 3 × 10^18^ cm^−3^, followed by an active region that consists of 76 cascade periods. The fine structure of one period can be found in the caption of Fig. 1. Finally, a 50-nm thick GaAs top contact layer with 5 × 10^18^ cm^−3^ doping is grown on top of the active region.

After the MBE growth, the wafer is processed into single plasmon waveguide ridge structures using standard laser processing technology. Optical lithography and an inductively coupled plasma (ICP) machine are used to define and etch the ridge structures. The top (Ti/Au, 10/300 nm) and lateral (Ge/Au/Ni/Au, 13/33/30/300 nm) metallic electrodes are fabricated employing electron-beam evaporation and a lift-off process. To form the ohmic contacts, the lateral metal is annealed at 370 °C for 40 s, and to improve the temperature performance of the devices, the semi-insulating substrate is thinned down to 150 *μ*m followed by a Ti/Au (10/300 nm) metal layer deposited on the backside. The fabricated ridge structures have a ridge width from 150 to 250 *μ*m, and the cavity length from 2 to 7 mm is defined by cleaving. The as-cleaved laser bar is indium-soldered on a copper heat sink. The electrical injection is achieved using wire bonding to connect the laser with the contact pads. To facilitate the RF modulation, a carefully designed 50-Ω microwave transmission line facing the back facet of the laser cavity is mounted behind the laser bar. Finally, the packaged device is screwed onto the cold finger of a liquid helium cryostat for low temperature measurements.

### Experimental characterizations

The cw power of the laser is measured using a terahertz power meter (Ophir 3A-P THz) with two parabolic mirrors for collecting and collimating the light onto the detection element. For simultaneously injecting DC and RF signal into the laser, a Bias-T with a bandwidth of 40 GHz is used. A microwave circulator is used for transmitting the RF signal into the Bias-T as well as directing the reflected inter-mode beat note signal into the spectrum analyser or oscilloscope. The beat note spectra are taken with a spectrum analyser, while the time domain traces are recorded using a high-speed oscilloscope together with a low-noise amplifier (30 dB gain). For the beat note mapping, a resolution bandwidth (RBW) of 300 kHz is used, while the RBW parameter used for the single-shot beat note spectra is dependent on the mode stability at various drive currents, normally ranging from 1 kHz to 10 kHz. All of the electronic equipments, RF cables and connectors are capable of working with at least 18 GHz frequency, which is enough sufficient for characterizing the inter-mode beat note signal at approximately 6.2 GHz. The terahertz emission spectra from free-running and RF-modulated lasers are measured using a FTIR spectrometer (Bruker v80). The experiment of RF modulation performed here is the same as the initial experiments done by S. Barbieri in ref. 15. The experimental setup is shown in Supplementary Fig. S1.

### Dispersion measurements

In the same manner as presented in ref. 7, to evaluate the dispersion of the terahertz QCL, we employ a zero-padding technique for the Fourier transform of the interferograms. The technique can smooth the spectra by generating more points in the curves and help us to accurately locate the mode peak frequencies and then derive the mode spacing. Because of this, the accuracy of the measured mode spacing is not limited by the FTIR spectral resolution. The interferograms were the same as those used in Fig. 4, and a zero padding with a zero filling factor of 256 was then added for the Fourier transform to interpolate the terahertz spectra. The residuals shown in Fig. 5 are defined as the differences between the measured peak frequencies and the predicted peak frequencies (the linear fit).

## Additional Information

**How to cite this article:** Wan, W. J. *et al*. Homogeneous spectral spanning of terahertz semiconductor lasers with radio frequency modulation. *Sci. Rep.* **7**, 44109; doi: 10.1038/srep44109 (2017).

**Publisher's note:** Springer Nature remains neutral with regard to jurisdictional claims in published maps and institutional affiliations.

## Supplementary Material

Supplementary Information
