# Supplementary material for: Homogeneous spectral spanning of terahertz semiconductor lasers with radio frequency modulation
Source: Sci Rep. 2017 Mar 8;7:44109. doi: 10.1038/srep44109 (PMC5341035; doi:10.1038/srep44109)
Supplement: Supplementary Information [file srep44109-s1.pdf]

<u>**Supplementary information:**</u>

Homogeneously spectral spanning of terahertz semiconductor lasers with radio frequency modulation

W. J. Wan, H. Li*, T. Zhou, and J. C. Cao*

Key Laboratory of Terahertz Solid-State Technology, Shanghai Institute of Microsystem and Information Technology, Chinese Academy of Sciences, 865 Changning road, Shanghai 200050, China

*Corresponding authors: hua.li@mail.sim.ac.cn and jccao@mail.sim.ac.cn

1. Experimental setup.

By employing the experimental setup as shown in Fig. S1, we are able to characterize the mode coherence of terahertz quantum cascade lasers (QCLs), achieve radio frequency (RF) modulation and terahertz emission spectra measurements. A broad bandwidth Bias-T is used to couple DC and RF signal simultaneously into the terahertz laser cavity. The reflected electrical beat note signal is directed by a circulator and then measured in frequency domain and time domain with a help of a microwave coupler. The beat note spectra are measured using a spectrum analyser while the time trace signal is measured using a high speed oscilloscope with assistance of a low noise amplifier (30 dB gain).

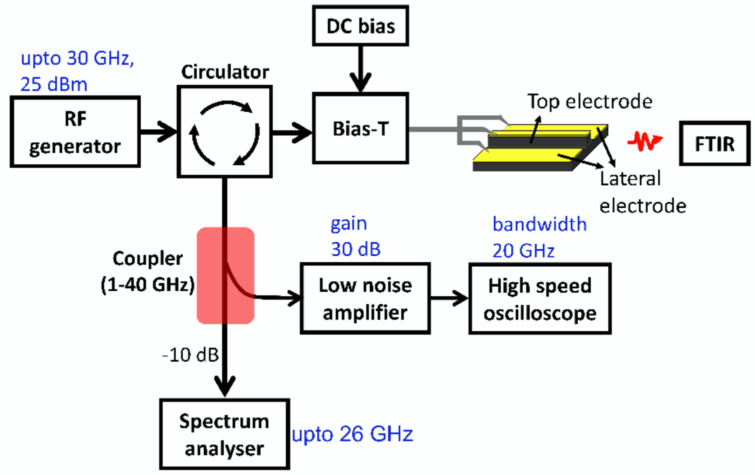

Figure S1. Experimental setup for the inter-mode beat note, RF modulation, and terahertz emission spectra measurements.

Concerning the Fourier Transform Infrared (FTIR) spectroscopy, a terahertz beam splitter (Mylar) and a far-infrared Deuterated TriGlycine Sulfate (DTGS) detector are used. The spectral resolution used in this work is 0.1 cm^{-1} (3 GHz). To get clean signal with high signal to noise ratio, the optical chamber of the FTIR is pumped down to 2 mbar and the THz beam path outside the FTIR is purged with dry air to reduce the water absorption as much as possible.

2. Short cavity length terahertz QCLs.

As we discussed in the main text, the 6-mm long terahertz QCL shows much better frequency stability than short cavity QCLs fabricated from the same MBE-grown wafer. Here in Fig. S2, we show the measured light-current-voltage (*L-I-V*) characteristics of a 2.5-mm long and 200-µm wide QCL in continuous wave (CW) mode at 10 K. Figure S3 shows the beat note evolution with drive current. In the current range investigated here the beat note frequency is around 14.8 GHz. We can clearly see that it shows

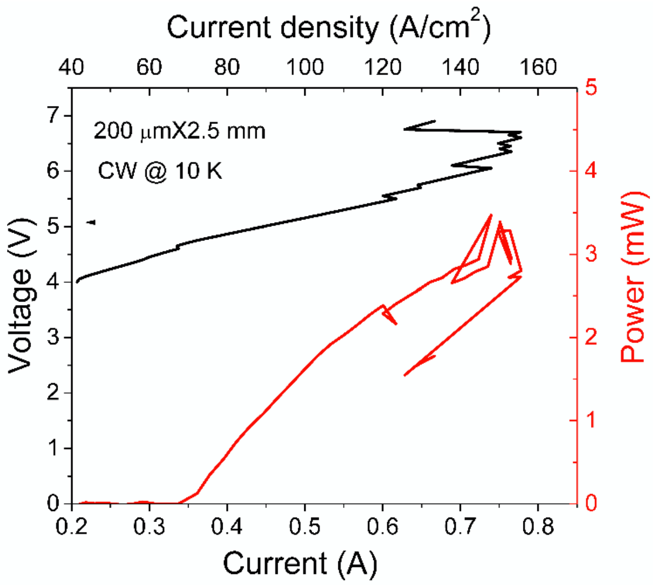

Figure S2. CW *L-I-V* characteristics of a 2.5-mm long terahertz QCL fabricated from the same wafer as the 6-mm long QCL reported in the main text.

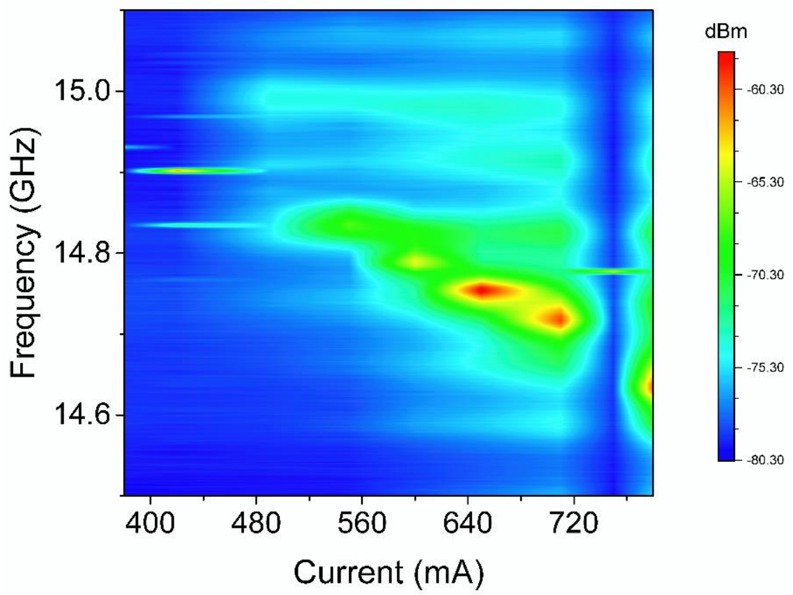

Figure S3. Beat note mapping of the 2.5-mm long QCL in free running.

completely different behavior from the 6-mm long laser. At most of currents, the short cavity laser presents broad beat note spectra which indicate large phase noise in the short cavity laser.

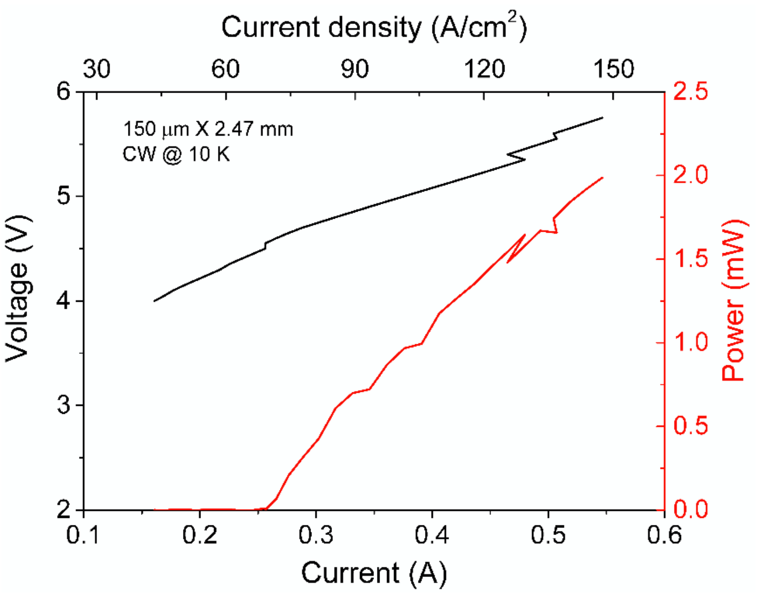

Figure S4. CW *L*-*I*-*V* characteristics of a 2.47-mm long terahertz QCL fabricated from the same wafer as the 6-mm long QCL reported in the main text.

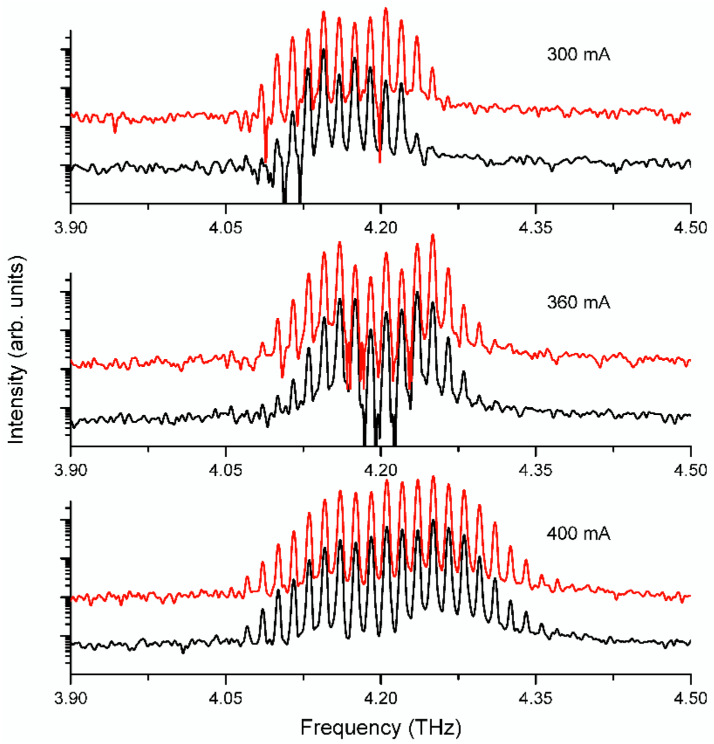

Figure S5. Comparison of the spectra of the 2.47-mm long laser working in free running mode (black) and under RF modulation (red). The red curves are vertically shifted for clarity. The RF power is 20 dBm.

In Figs. S4 and S5, we show the experimental results of another short cavity laser with a cavity length of 2.47 mm and ridge width of 150 µm. From Figs. S2 and S4, we find the laser threshold and maximum current density agree well with each case. We therefore can infer a high uniformity of wafer quality and the laser processing technology. Figure S5 shows the terahertz emission spectra without (black) and with (red) RF modulation at peak frequency of the free running beat note spectrum. We can see that at various currents, the RF modulation cannot significantly affect the emission spectra of the short cavity laser. In some cases, we just get 1 or 2 more modes appeared with the strong RF modulation.

Figures S3 and S5 demonstrate that the short cavity laser has worse mode coherence and therefore it is more difficult to be effectively modulated with RF. In Fig. S6 we show another factor that could influence the RF modulation. What we did is the following: we inject RF signal with different frequencies but constant power of 25 dBm as the dashed line shows in Fig. S6, to a 1.5-m long RF cable from one end. And then we measured the transmitted power at the other end. For all the RF frequencies, we can observe big transmission attenuations. However, as the frequency is increased, the attenuation increases too. If we compare the attenuations at 6 and 15 GHz, almost 4 dB difference can be verified. We therefore can conclude that it is less effective to inject higher frequency RF signal into the laser cavity simply due to the natural cable attenuation.

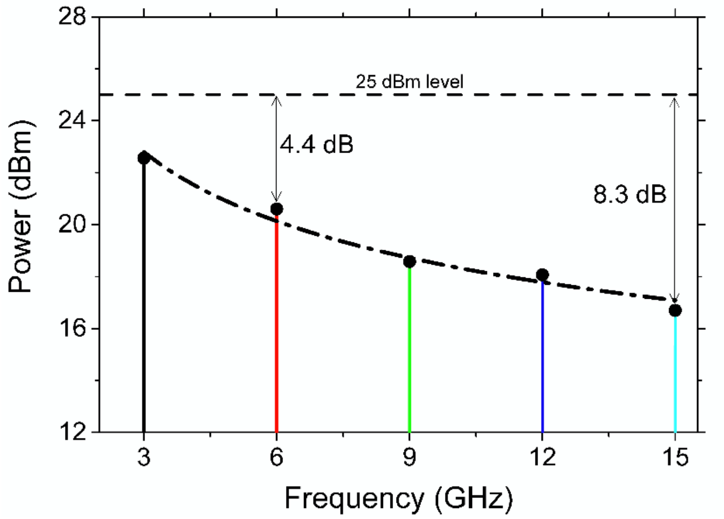

Figure S6. Frequency dependence of microwave signal attenuation via a 1.5-m long RF cable.
